# Analysis of clinical phenotypes and genotypes of congenital deafness caused by rare variants in *GJB2*

**DOI:** 10.3389/fped.2025.1514369

**Published:** 2025-04-23

**Authors:** Xuxu Zhao, Huan Chi, Yan Bai, Yu Lu, Wenyu Xiong, Houyong Kang, Cheng Zhang

**Affiliations:** ^1^ Department of Otorhinolaryngology, Head and Neck Surgery, The First Affiliated Hospital of Chongqing Medical University, Chongqing, China; ^2^ Department of Otolaryngology, National Clinical Research Center for Child Health and Disorders, Ministry of Education Key Laboratory of Child Development and Disorders, Chongqing Key Laboratory of Pediatric Metabolism and Inflammatory Diseases, Chongqing Key Laboratory of Child Rare Diseases in Infection and Immunity, Chongqing Key Laboratory of Child Neurodevelopment and Cognitive Disorders, Chongqing Key Laboratory of Structural Birth Defect and Reconstruction, Chongqing Engineering Research Center of Stem Cell Therapy, Children’s Hospital of Chongqing Medical University, Chongqing, China; ^3^ West China Hospital, Sichuan University, Sichuan, China; ^4^ Department of Otorhinolaryngology, Head and Neck Surgery, The Second Affiliated Hospital of Chongqing Medical University, Chongqing, China

**Keywords:** *GJB2*, hereditary deafness, genotype-phenotype association, clinical characteristic analysis, congenital deafness

## Abstract

**Objective:**

This study aims to analyze a genetic family with the *GJB2* gene c.551G>A (p.R184Q) variant, exploring the relationship between its genotype and clinical phenotype, and summarizing the inheritance pattern and clinical features associated with this locus.

**Methods:**

Detailed medical history collection and physical examinations were conducted for the proband and their family members. Audiological assessments and genetic sequencing analyses were performed on some members. Additionally, a review of existing literature concerning GJB2 c.551G>A (p.R184Q) was conducted.

**Results:**

The proband, along with their father and paternal grandmother, carried the heterozygous mutation GJB2 c.551G>A, all exhibiting moderate to profound bilateral prelingual sensorineural deafness. Notably, the proband also presented symptoms of skin dryness and nail abnormalities characteristic of syndromic hearing loss.

**Conclusion:**

The GJB2 c.551G>A mutation not only leads to severe hearing loss but may also be associated with syndromic hearing loss, expanding our understanding of the clinical spectrum associated with this variant.

## Introduction

Hearing loss due to genetic factors is a major cause of hearing impairment in newborns. Recent epidemiological studies indicate that half of all newborn hearing losses can be attributed to genetic factors (1). Genetic hearing loss is categorized into syndromic and non-syndromic types. Currently, over 150 genes associated with hearing loss have been reported (2). *GJB2* is the most common gene responsible for deafness in Chinese newborns (3). The discovery of rare variants within the *GJB2* gene has marked a significant milestone in unraveling the genetic underpinnings of congenital deafness. *GJB2* is responsible for producing connexin 26, a protein essential for the creation of gap junctions within the cochlea. Alterations in this gene can disrupt the normal operation of these junctions, causing hearing loss. Such mutations can give rise to a spectrum of genetic patterns and phenotypes, including autosomal recessive non-syndromic hearing loss, autosomal dominant non-syndromic hearing loss, and syndromic hearing loss (4). Syndromic hearing loss often presents when the auditory system is compromised in conjunction with the integumentary system, with related syndromes encompassing Keratitis-Ichthyosis-Deafness syndrome (KID), and Palmoplantar Keratoderma (PPK) linked to deafness (5, 6). Skin phenotypes associated with syndromic hearing loss due to *GJB2* mutations encompass ichthyosis, palmoplantar keratoderma, joint pads, and nail abnormalities (7). The c.551G > A (p.R184Q) variant of the *GJB2* gene is a rare mutation with limited documentation and some debate surrounding it. Nonetheless, the clinical phenotypes and genotypes linked to these rare variants are intricate and exhibit variability, necessitating a thorough analysis to clarify the underlying mechanisms.

In our study, we performed a comprehensive analysis of a genetic family to uncover the connection between the *GJB2* c.551G>A mutation and syndromic hearing loss, concentrating on the phenotypic traits and inheritance patterns observed within this family. Our results indicate that the *GJB2* c.551G>A mutation significantly contributes to the development of syndromic hearing loss in this genetic cohort, offering new insights that could enhance the diagnosis and genetic counseling for hereditary deafness.

### Subjects and methods

**Figure 1:**
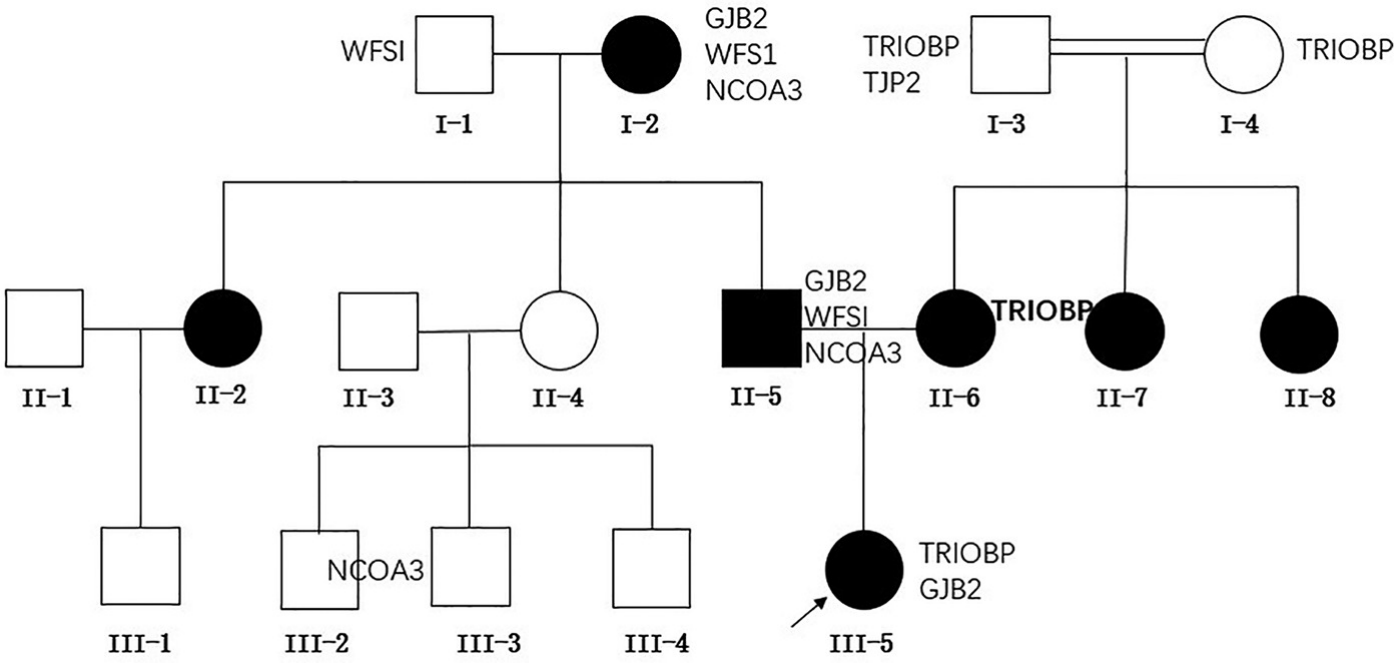
Pedigree and the genotypes of family members. Bold indicates homozygous.

This study conducted an analysis on the family of a patient who had been deaf from birth, diagnosed with severe bilateral hearing impairment at the age of one at the Children's Hospital of Chongqing Medical University. The family tree is depicted in Figure 1. This study was approved by the Ethics Committee of Children's Hospital of Chongqing Medical University (Number 2024-383). This study adhered strictly to the ethical guidelines outlined in the Declaration of Helsinki and its subsequent amendments. Given the retrospective nature of the research, the institutional review board granted permission to dispense with the requirement for written informed consent.

### Genomic DNA extraction and gene sequencing

Upon securing informed consent from the participants' family members, genomic DNA is extracted from peripheral venous blood samples. Utilizing whole-exome sequencing (WGS), the *GJB2* gene and associated genes are sequenced to identify potential pathogenic mutations.

### Medical history collection and physical examination

We perform a thorough collection of medical histories for patients with a family history of hearing loss, encompassing past instances of otitis media, meningitis, mumps, head injuries, a history of ototoxic medication use, maternal exposure to risk factors during pregnancy, and occurrences of hyperbilirubinemia, asphyxia, or hypoxia at birth. The physical examination zeroes in on evaluating the skin for signs of thickening, dryness, peeling, keratosis, erythema, or calluses, and also involves inspecting the nails for irregularities such as dysplasia, deep grooves, thickening, brittleness, or koilonychia, while also verifying the presence of digital pads without keratitis.

### Audiological examination

The audiological examination includes otoscopic evaluation, pure tone audiometry (PTA), auditory brainstem response (ABR), distortion product evoked otoacoustic emissions (DPOAE), and tympanometry. Hearing impairment severity is categorized according to PTA outcomes, with classifications ranging from mild (20–40 dB), moderate (41–70 dB), severe (71–95 dB), to profound (>95 dB).

## Results

### Clinical manifestations of proband and family members

**Figure 2:**
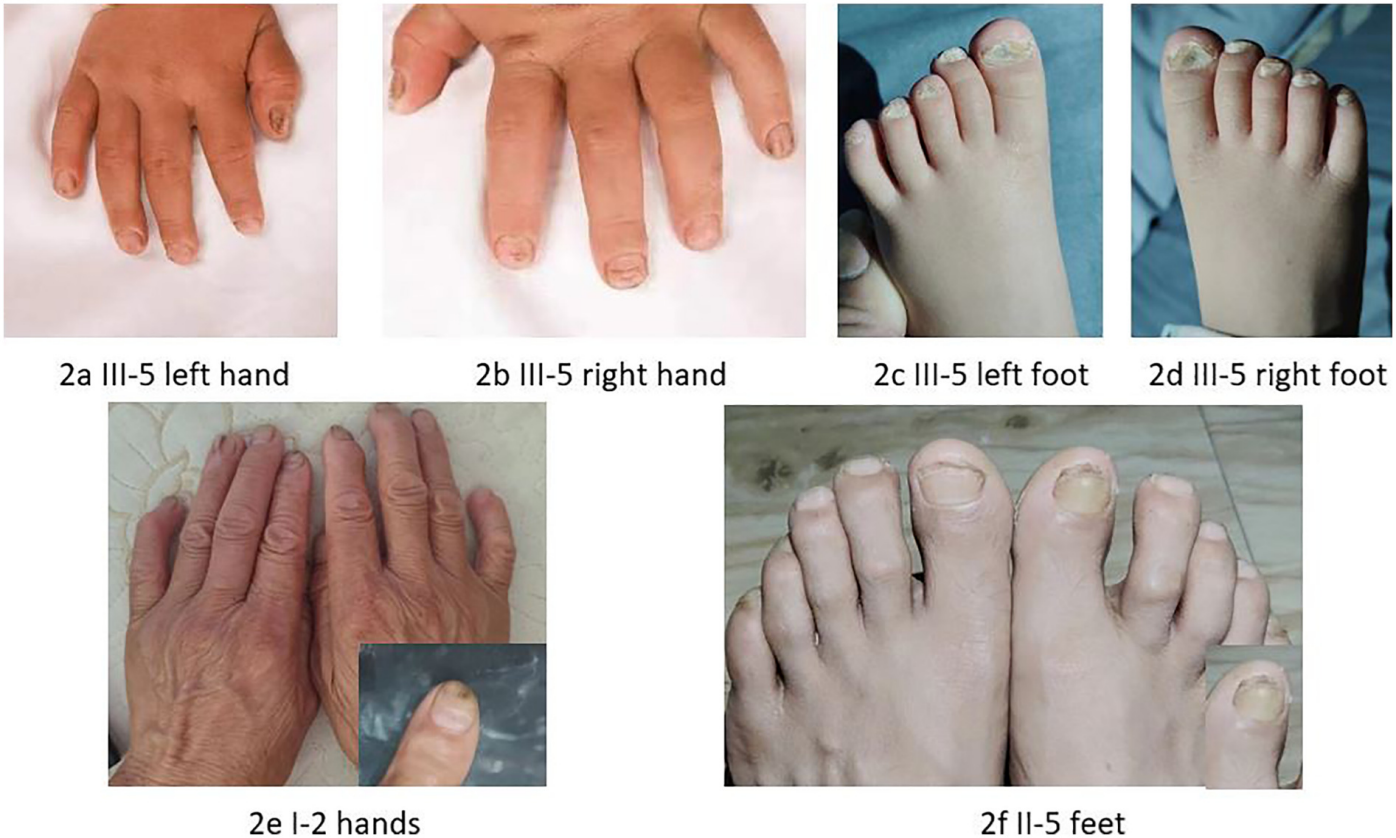
Phenotypic characterization of selected family members. **(a,b)** The proband's hands exhibited thickened fingernails, some of which showed a dimple. **(c,d)** The proband's feet displayed similarly thickened and keratotic toenails. **(e)** The hands of the proband's grandmother showed a dimple on the left index finger (enlarged detail in lower right corner). **(f)** The feet of the proband's father exhibited thickening and hyperpigmentation at the distal end of the right great toenail (enlarged detail in lower right corner).

**Figure 3:**
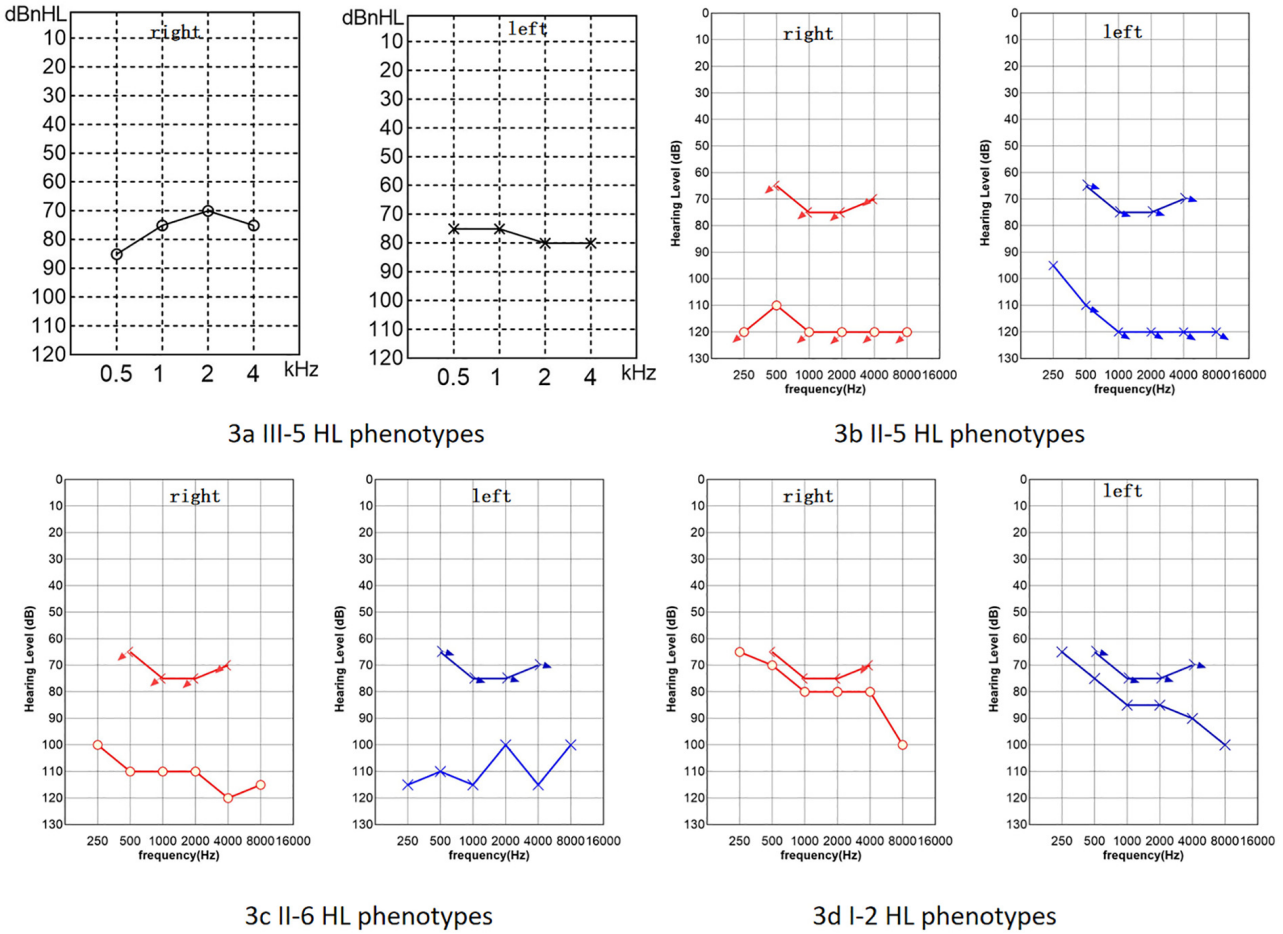
Hearing loss (HL) phenotypes of some family members. **(a)** The proband's ASSR. **(b)** PTA of the proband's father. **(c)** PTA of the proband's mother. **(d)** PTA of the proband's grandmother. Red circles represent air conduction audiometry for the right ear, blue Xs represent air conduction audiometry for the left ear, brackets represent bone conduction audiometry. The downward arrow indicates no response at the maximum audiometer output value **(b–d)**.

The proband was a 1-year-old female patient with no history of otitis media, meningitis, mumps, or head trauma. She had not been exposed to ototoxic drugs, and her mother had no exposure to risk factors during pregnancy. At birth, there were no instances of hyperbilirubinemia, asphyxia, or hypoxia. Cytomegalovirus screening was negative. No obvious abnormalities were found in the proband's ECG, urinalysis, or abdominal ultrasound. The family denied that the child had vision loss, double vision, or other problems with their eyes. The patient exhibited thickened, keratotic fingernails and toenails, with some having a spoon-shaped appearance (Figures 2a–d), and had dry skin on her face and feet, We invited a dermatologist to examine the proband's skin and nails during her hospital stay. After examining the patient, the dermatologist considered genetic factors. Auditory brainstem response (ABR) testing revealed air conduction was 95 dB nHL in the left ear and 95 dB nHL in the right ear, while bone conduction did not respond at 50 dB nHL in both ears. The auditory steady-state response (ASSR) was examined at nine months, as depicted in Figure 3a, and transient evoked otoacoustic emissions (TEOAE) were not elicited. No significant abnormalities were detected in acoustic immittance, otoscopy, inner ear MRI, or inner ear CT. The proband attempted to use a hearing aid but did not respond to it. At the age of two, she underwent cochlear implantation and is still undergoing speech rehabilitation.

The proband's paternal grandmother (I-2) has had poor hearing since childhood, with PTA results shown in Figure 3d, which indicates severe sensorineural deafness in both ears. She can comprehend common words in daily conversation, but struggles with long sentences. Her coherent speech is not intelligible, yet with context and lip-reading cues, her family can gradually understand individual words in her speech. There are no signs of skin thickening or dryness, but she reports cracks in the nail of her left ring finger following an injury, and a dimple is visible on her left index finger (Figure 2e). The proband's father (II-5) has PTA results indicating profound deafness (Figure 3b), and family members report poor sound response following high fever at ages 5–6, He is unable to respond to sounds in daily life; his words are not easily recognized. He communicates through gestures. The distal end of his right big toe nail is thickened and darkened (Figure 2f). The proband's mother (II-6) also shows profound deafness according to audiometry results (Figure 3c), She is unresponsive to sounds in her daily environment and unable to articulate meaningful words.Neither I-2, II-5, nor II-6 has a history of otitis media, meningitis, mumps, or head trauma, and no significant skin abnormalities were observed.

### Genetic sequencing results

Genetic sequencing has uncovered that the proband possesses a heterozygous *GJB2* c.551G>A mutation, aligning with the genotypes of the proband's father and paternal grandmother. Furthermore, the proband harbors a heterozygous *TRIOBP* c.2176C>T mutation, whereas the mother displays a homozygous mutation at this locus, and both the maternal grandfather and grandmother carry heterozygous mutations at this site.

### The association between genotype and phenotype in this lineage

Through a detailed analysis of the genotypes and phenotypes of family members, we found a strong association between the *GJB2* c.551G>A mutation and the hereditary deafness phenotype within the family. Furthermore, the *TRIOBP* c.2176C>T mutation was also detected in the mother of the affected individual and additional relatives, aligning with the characteristics of autosomal recessive non-syndromic deafness.

### Summary of case characteristics for *GJB2*: c.551g>A (p.R184q)

We summarized previous reports related to the *GJB2* c.551G>A (p.R184Q) locus (Table 1), finding a total of 9 publications covering 20 patients, of which only one was diagnosed with syndromic deafness. All known auditory phenotypes were prelingual, ranging from severe to profound hearing loss. In some cases, the pathogenic gene occurred *de novo*, and all known inheritance patterns are autosomal dominant.

**Table 1:** Review of the literature on GJB2 c.551G > A(p.R184Q).

Number	Year	Country	Sex	Genotype	Origin	Phenotype		
						Prelingual	Degree^4^	Abnormal skin
1	2001 (12)	Ghana	female	c.551G > A/WT	-	-	-	no
2			female	c.551G > A/WT	mother	-	-	no
3			male	c.551G > A/WT	mother	-	-	no
4			male	c.551G > A/WT	mother	-	-	no
5			male	c.551G > A/WT	mother	-	-	no
6	2002 (21)	China	male	c.551G > A/WT	*de novo* ^5^	-	-	no
7	2010 (11)	Iranian	male	c.551G > A/WT	*de novo*	yes	severe	no
8	2011 (10)	China	-	c.551G > A/WT	*de novo*	yes	profound	no
9			-	c.551G > A/WT	*de novo*	yes	profound	no
10	2014 (13)	China	female	c.551G > A/WT	*de novo*	yes	severe	yes^1^
11			-	c.551G > A/WT	*de novo*	yes	profound	no
12	2017 (9)	Indian	male	R184Q/Q124X/IVS1 + 1G^3^	-	yes	profound	no
13			female	R184Q/Q124X/IVS1 + 1G	father	yes	profound	no
14			female	R184Q/Q124X/IVS1 + 1G	-	yes	Moderate- severe^2^	no
15	2020 (22)	China	female	c.551G > A/WT	-	yes	profound	-
16			female	c.551G > A/WT	mother	yes	profound	-
17	2022 (23)	Morocco	male	c.551G > A/WT	-	-	severe	no
18			male	c.551G > A/WT	father	-	severe	no
19			male	c.551G > A/WT	father	-	severe	no
20	2024 (8)	Morocco	-	c.551G > A/WT	-	yes	-	no

(1) Thickening and peeling of the skin (soles), keratoderma (soles), callus (soles), thickened nails (fingers), spoon nails (toes). (2) Moderate in the right ear and severe sensorineural hearing loss(SNHL) in the left ear. (3) Triallelic combination. (4) Hearing loss degree: mild (21–40 dB), moderate (41–70 dB), severe (71–95 dB), and profound (>95 dB). (5) His father was deaf, but he did not possess the same mutation.

## Discussion

### The clinical significance of the *GJB2* c.551g>A mutation

In this study, we document a family affected by *GJB2*-related prelingual severe to profound hearing loss, in which the proband also manifests characteristics of syndromic hearing impairment, including xeroderma and nail dystrophy. The GJB2 c.551G>A variant is a rare dominant mutation. As far as we are aware, this variant has been reported in only five countries: Ghana, Iran, China, India, and Morocco (8–12). Up to now, the GJB2 c.551G>A variant has been linked to autosomal dominant non-syndromic hearing impairment, with only a single case previously reported involving autosomal dominant syndromic hearing loss. The proband exhibits skin symptoms that are consistent with those described in a patient reported by Xiuhong Pang (13), who had a syndromic GJB2 c.551G>A heterozygous mutation. Our findings suggest that the GJB2 c.551G>A variant can result in both syndromic and non-syndromic forms of hearing loss.

*GJB2* is located on chromosome 13q11-12 and encodes the human connexin 26 protein, a member of a multigene family comprising 20 structural proteins. This protein is extensively expressed across numerous human tissues, encompassing epithelial cells originating from the cochlear ectoderm, the cornea, and the skin. The *GJB2* c.551G>A mutation represents a missense alteration, causing the replacement of glutamine with arginine at amino acid position 184. Research has shown that connexins with this mutation are incapable of forming functional gap junction channels (14). Furthermore, experiments conducted on HeLa cells have revealed that the mutated R184Q protein exerts a dominant negative impact on both wild-type connexin 26 and connexin 30 (15). Studies indicate that Cx26 mutations contribute to the development of various conditions by promoting cell death or exerting a dominant negative effect on co-expressed connexins, which can lead to skin disorders and hearing loss (16). Conversely, mutations that diminish channel function may exclusively result in hearing loss. Consequently, from a mechanistic standpoint, *GJB2*: c.551G>A is likely to induce syndromic hearing impairment.

The phenotypes of *GJB2*-related deafness are diverse, encompassing both syndromic and non-syndromic forms, with many locations being quite common. Patients with *GJB2* p.R75Q-associated hearing loss may present with skin abnormalities or have no obvious skin conditions (17). The GJB2 c.250G>A variant is generally thought to be associated with post-lingual, severe to profound hearing loss, but there have been reported that this mutation lead to mild hearing loss and skin keratoderma syndromic deafness (18). Moreover, the skin phenotype in individuals with dominant syndromic deafness related to *GJB2* varies in severity among different individuals. In the family we studied, the proband exhibited generalized dry skin and spoon-shaped nails, presenting more severe symptoms than their father and paternal grandmother, who shared the same genotype. Several speculations may explain this observation: (1) Additional heterozygous recessive mutations may alter the hearing loss and skin phenotype caused by the dominant *GJB2* mutation. Research indicates that family members with p.R75W/c.235delC, where *GJB2* c.235delC can cause autosomal recessive non-syndromic deafness, manifest more severe hearing loss and palmoplantar keratoderma than those with only the p.R75W mutation (13). (2) The influence of environmental and ethnic factors. The proband is only 1 year old and could not complete subjective hearing tests, so auditory evaluation was performed using ASSR. The father and grandmother underwent subjective hearing assessments, and the degree of hearing loss could not be compared. The hearing loss in II-5 is more severe than in I-2. We learned that II-5 experienced a severe fever at ages 5–6, after which he showed a poor response to sound. Whether environmental factors exacerbate *GJB2*-related deafness requires further validation in the future.

### Diversity of genetic patterns

In our study, the pathogenic genes of the proband and the proband's father both originated from the grandmother of the proband. The variation at this locus exhibits an autosomal dominant inheritance pattern, consistent with previous reports. However, some studies suggest that mutations at this locus may be *de novo* mutations. One family reported by Amritkumar Pavithra includes three patients: a brother and sister, along with the sister's daughter, all of whom have compound heterozygous mutations, along with mutations in other recessive genes. Their parents have normal hearing, making it impossible to completely rule out the possibility of a gonadal mosaicism in either parent as the source of the P.R184Q mutation in the context of autosomal dominant inheritance (9). This diversity in inheritance patterns emphasizes the importance of considering multiple genetic mechanisms in hereditary hearing loss research.

### Potential impact of TRIOBP: c.2176c>T mutation

Mutations in *TRIOBP* can cause autosomal recessive nonsyndromic deafness, which is associated with prelingual, severe to profound hearing loss (19). Very few mutations in the site manifest as postlingual deafness. More than 40 loci have been reported to cause disease in this gene (20). The proband's mother had profound sensorineural deafness and was homozygous for *TRIOBP* c.2176C>T. Both of her parents were heterozygous for *TRIOBP* c.2176C>T and were consanguineous. Her two younger sisters also had prelingual deafness. We were unable to obtain the results of genetic and hearing tests for her two younger sisters. There are no relevant literature reports on mutations in this site. We only found one record in the ClinVar database that mutations in this site may cause disease. The family we reported provided strong evidence that the *TRIOBP* c.2176C>T mutation caused autosomal recessive nonsyndromic deafness, which was associated with prelingual and profound hearing loss, and broadened the spectrum of pathogenic mutations in the *TRIOBP* gene.

### Future research directions

The clinical phenotype of *GJB2*-related dominant syndromic deafness varies in severity among different individuals. In the same family, the same mutation site may cause different degrees of hearing loss and skin damage, and the mechanism behind it deserves further exploration. Furthermore, the two documented cases of syndromic deafness associated with the *GJB2* c.551G>A mutation are both individuals of Chinese Han ethnicity. Whether this form of dominant syndromic deafness linked to *GJB2* c.551G>A is exclusive to this demographic remains to be determined. Future research should delve into the prevalence and phenotypic variability of the *GJB2* c.551G>A mutation across various populations. Concurrently, the functional implications of the *TRIOBP* c.2176C>T mutation and its role in causing deafness merit further investigation.

## Conclusion

Our study reported a pedigree with syndromic deafness caused by the *GJB2* p.R184Q mutation, summarized the phenotype and inheritance mode of *GJB2* p.R184Q reported previously, and confirmed that *GJB2* p.R184Q is associated with both syndromic and non-syndromic deafness. By understanding the genetic underpinnings and associated clinical presentations, we can improve diagnostic accuracy, provide more personalized management strategies, and ultimately enhance the quality of life for individuals affected by this form of hearing impairment. Future research should continue to unravel the intricate interplay between genetic variants and environmental factors to further advance our knowledge and treatment options for *GJB2*-related deafness.

## Data Availability

The original contributions presented in the study are included in the article/Supplementary Material, further inquiries can be directed to the corresponding author.
